# SG-APSIC1206: Effectiveness of sterilization practice in reprocessing medical devices among different multidisciplinary tertiary-care hospitals in Dhaka City

**DOI:** 10.1017/ash.2023.97

**Published:** 2023-03-16

**Authors:** Sifat Uz Zaman, Nihad Adnan

**Affiliations:** Jahangirnagar University, Savar, Bangladesh; Jahangirnagar University, Savar, Bangladesh

## Abstract

**Objectives:** Sterilization failure is one of the main causes of surgical-site infections. We assessed the effectiveness of the sterilization process of surgical instruments to determine the reasons for sterilization failure. **Methods:** In total, 100 sterilization cycles were observed from February 4, 2022, to September 5, 2022, in hospitals in Dhaka City. We used sterilization quality assurance monitoring tools (ie, biological indicators) for rapid steam and ethylene oxide sterilization methods. Tests were performed using an automatic reading machine, chemical indicator strips, and indicator tape for both steam and ethylene oxide methods. For laboratory testing and data collection, APSIC guidelines were followed. All samples were incubated for 48 hours to cross check the accuracy of the auto-reader result. **Results:** All ethylene oxide sterilization cycles were 100% successful, as shown by the rapid biological indicator (auto-reader), chemical indicator strips, and indicator tape. However, 22% sterilization failure occurred with steam sterilization, which was confirmed by the auto-reader, chemical indicator strips, and indicator tape. All biological samples showed no growth after 48 hours of incubation, except the sample from steam sterilization, which did show growth after 48 hours of incubation. **Conclusions:** We detected 22% steam sterilization failure, and serious harm to patients could occur if these surgical instruments were used for surgery. Process recall would not have been not possible if rapid biological indicator tests had not been performed and other chemical monitoring tools had not been used. The regular use of monitoring tools according to guidelines can be a reliable solution to reduce surgical site infections caused by inappropriate sterilization of surgical instruments.

